# *Klebsiella pneumoniae* in the intestines of *Musca domestica* larvae can assist the host in antagonizing the poisoning of the heavy metal copper

**DOI:** 10.1186/s12866-023-03082-7

**Published:** 2023-12-04

**Authors:** Yansong Yin, Shumin Wang, Kexin Zhang, Ying Li, WenJuan Liu, Qian Zhang, Xinyu Zhang, Xinxin Kong, Sha An, Ruiling Zhang, Zhong Zhang

**Affiliations:** 1 School of Basic Medical Science, Shandong First Medical University (Shandong Academy of Medical Sciences), Taian, 271016 Shandong China; 2 Collaborative Innovation Center for the Origin and Control of Emerging Infectious Diseases, Shandong First Medical University (Shandong Academy of Medical Sciences), No. 619, Changchen Road, Taian, 271016 Shandong China; 3 School of Life Science, Shandong First Medical University (Shandong Academy of Medical Sciences), Taian, 271016 Shandong China; 4 Weifang Medical University, Weifang, 261021 Shandong China

**Keywords:** Cu^2+^, *Musca domestica* larvae, *Klebsiella pneumoniae*, High-throughput sequencing

## Abstract

**Background:**

*Musca domestica* larvae are common saprophytes in nature, promoting the material—energy cycle in the environment. However, heavy metal pollution in the environment negatively affects their function in material circulation. Our previous research found that some intestinal bacteria play an important role in the development of housefly, but the responses of microbial community to heavy metal stresses in *Musca domestica* is less studied.

**Results:**

In this study, CuSO_4_, CuSO_4_—*Klebsiella pneumoniae* mixture and CuSO_4_—*K. pneumoniae* phage mixture were added to the larval diet to analyze whether *K. pneumoniae* can protect housefly larvae against Cu^2+^ injury. Our results showed that larval development was inhibited when were fed with CuSO_4_, the bacterial abundance of *Providencia* in the intestine of larvae increased. However, the inhibition effects of CuSO_4_ was relieved when *K. pneumoniae* mixed and added in larval diets, the abundance of *Providencia* decreased. Electron microscope results revealed that *K. pneumoniae* showed an obvious adsorption effect on copper ion in vitro.

**Conclusions:**

Based on the results we assume that *K. pneumoniae* could adsorb Cu^2+^, reduce Cu^2+^ impact on gut community structure. Our study explains the role of *K. pneumoniae* antagonizing Cu^2+^, which could be applied as a probiotic to saprophytic bioantagonistic metal contamination.

## Background

In recent years, ecological and global public health issues associated with metal environmental pollution have received increasing attention [1]. When certain metals like iron (Fe), copper (Cu), nickel (Ni) and zinc (Zn) exceed certain thresholds, they are toxic to organisms [2]. Copper (Cu) is a crucial trace element for poultry and ruminant production and involves various physio-chemical or chemico-physiological processes and metabolisms in animal growth [3]. Copper sulfate (CuSO_4_) is commonly used as an additive in animal feed to promote growth and prevent copper deficiencies in the animals. It can also be used as a disinfectant to prevent the occurrence of diseases [4]. However, over-application of Cu^2+^ as a feed additive in poultry and livestock production systems would result in excessive Cu^2+^ entering the environment. Research has shown that Cu, Zn, Cd, Pb, Cr and Ni have been detected in poultry and livestock manure [5], and some of which even exceed the national heavy metal limit for animal feed [6]. Due to their bioaccumulation and nondegradable property, these metals combined with animal waste and urine to form organic compost. Heavy metals cannot be degraded during composting [7], and eventually, Cu may accumulate in aquatic foods, plants (fruits, crops, and vegetables), and drinking water and endanger human health [8].

With the rapid development of poultry and livestock farming around the world, biotransformation of saprophytic insects has emerged as a promising approach for sustainable livestock and poultry manure management in addition to traditional manure treatment techniques [9]. *M. domestica* larvae are resource-based insects that thrive in feces and organic matter. It can be used as an efficient method for the biotransformation of pig manure and is regarded as a sustainable substitute for pig manure management [10]. However, heavy metal residues in feces have adverse effects on larval growth and biological functions of their transformation ability [11]. Therefore, it is of great importance to investigate toxicity mechanism of heavy metals and the adaptation mechanism of *M.domestica* larvae for improving the efficiency of *M.domestica* larval biotransformation.

The heavy metals in the environment enter the insect through food intake and mainly accumulate in the insect intestine. Insects harbor diverse microorganisms in the gut, complex environments where gut microbes interact and compete with microorganisms from the outside world, providing their host with physiological and ecological advantages [12, 13]. Gut microbiota, especially some beneficial flora, play an important role in maintaining the normal physiological function of the host [14]. However, how the gut microbial community responses to heavy metal and the role specific bacteria played when *M.domestica* larvae encountered heavy metal stress has been little studied.

Some studies have shown that metals have a significant effect on the intestinal community structure of insects, fish and animals. Exposure to cadmium can lead to a decrease in the abundance of *Bifidobacteria* and *Prevotella* in the intestine, thereby leading to lipid metabolism disorders and fat accumulation in the liver of mice [15]. The intestinal flora of *Hermetia illucens L.* exposed to Cu^2+^ and Cd^2+^ changed significantly [16]. Honeybees subtly change the overall composition of the microbiome and metabolic group of honeybees after exposure to Cd^2+^ [17]. Cd^2+^ treatment significantly changed the richness and diversity of the microbiota in zebrafish and was reported to be harmful to zebrafish health by altering its gut community structure [18]. The relative abundance of *Aeromonas* decreased in the intestinal community structure of *Bufo gargarizans tadpoles* after exposed to Cr^2+^ indicating that *Aeromonas* were susceptible to Cr^2+^ stress [19]. At present, there is a lack of detailed reports on the toxicity mechanism of heavy metals and their interactions with intestinal flora on houseflies.

Our previous studies have shown that intestinal probiotics in *M.domestica* larvae can promote larval growth. *Klebsiella pneumoniae* is one of our identifed beneficial bacteria that is essential for the growth and development of *M.domestica* larvae [20]. Therefore, in this paper, we analyzed the effects of heavy metals on the gut flora of houseflies and the beneficial bacteria *K. pneumoniae* played when larvae face heavy metal stress.

The interactions between *K. pneumoniae* and Cu^2+^ was investigated, and the microecological mechanism of *K. pneumoniae* antagonizing metals was further analyzed. We found that when *M.domestica* larvae encounter Cu^2+^ stress, *K. pneumoniae*, as a beneficial bacterium in the intestinal community, can alleviate the host damage caused by Cu^2+^ through adsorption of Cu^2+^. An antagonistic relationship between the beneficial bacteria in the gut of *M.domestica* larvae and heavy metals was found in this study, which provided a new possibility for the control of heavy metal pollution in the ecological environment.

**Reasults**

### Effects of the interaction of Cu^2+^—*Klebsiella pneumoniae *and its phage mixture on the growth and development of *Musca domestica* larvae

**Fig. 1:**
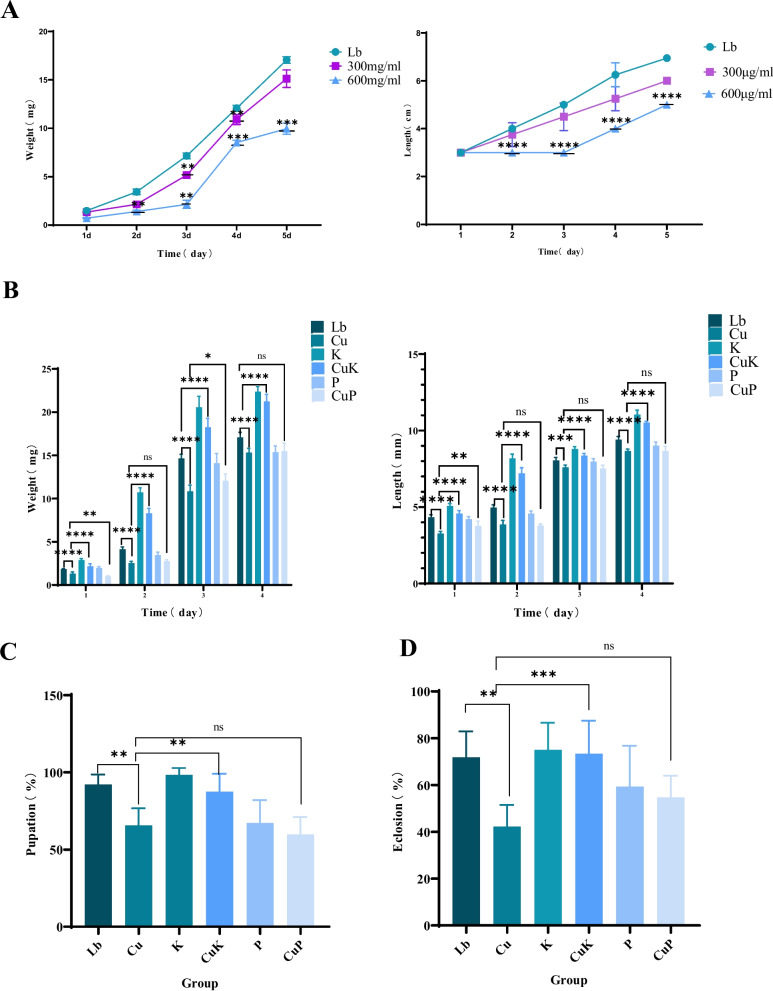
Effects of different groups on the growth and development of housefly larvae. **A** The body weight and body length of housefly larvae fed 300 μg/mL and 600 μg/mL CuSO_4_. **B** The body weight and body length of housefly larvae in different experimental groups. **C** Pupation rate in different experimental groups. **D** Eclosion rate of different experimental groups Abbreviations: Lb, Luria–Bertani medium; Cu, CuSo_4_; K, *K. pneumoniae* solution; CuK, Mixed solution of *K. pneumoniae* and CuSO_4_; P, *K. pneumoniae* phage; CuP, Mixed solution of *K. pneumoniae* phage and CuSO_4_. Asterisks indicate significant differences at *p < 0.05, **p < 0.01, ***p < 0.001,****p < 0.0001

In order to verify the interaction between metals and *K. pneumoniae* in the organism, we added Cu^2+^—*K. pneumoniae* to the *M.domestica* larvae and observed their effects on the growth and developmental ability. Based on the previous investigations [21–23], the common range of metal content of copper (Cu) was detected in the feces of poultry and livestock and within this range 300 μg/mL and 600 μg/mL of CuSO_4_ were selected for the subsequent experiments.After analysis, we found that Cu^2+^ inhibited the growth and development of *M.domestica* larvae and died at 600 µg/mL (Fig. 1A). Therefore, we selected 300 µg/mL CuSO_4_ for follow-up experiment. The results showed that the body weight (14.1 mg) and length (8 mm) of *M.domestica* larvae fed CuSO_4_ (Cu) were significantly lower than those of the control group (Lb). The growth and development of *M.domestica* larvae exposed to 300 μg/mL were significantly inhibited (Fig. 1B), and the pupation rate was significantly reduced (Fig. 1C). The body weight and length of *M.domestica* larvae fed CuSO_4_—*K. pneumoniae* (CuK) were significantly higher than those of the Lb group. Compared with the Cu group, the body weight increased significantly and the inhibition of *M.domestica* larval development was significantly alleviated (Fig. 1B), and the pupation rate and eclosion rate increased significantly (Fig. 1 C and D). When CuSO_4_—*K. pneumoniae* and its phage were fed together (CuP), the body weight and length of *M.domestica* larvae were significantly reduced compared with Lb group (Fig. 1B). Compared with Cu group, there was no significant difference in pupation rate and eclosion rate of *M.domestica* larvae (Fig. 1 C and D).

### Effects of the interaction of the heavy metal Cu^2+^—*K. pneumoniae *and its phage mixture on the creeping ability of *M. domestica* larvae

**Fig. 2:**
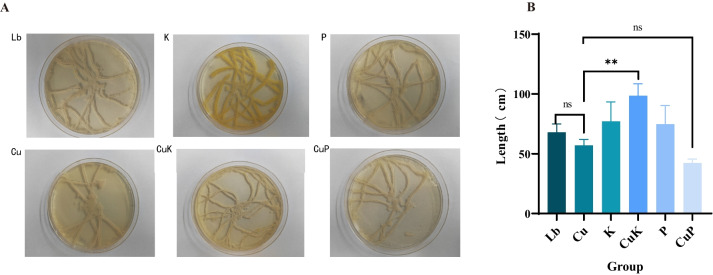
The creeping ability of housefly larvae fed with different groups. **A** The creeping trajectory of housefly larvae in solid agar medium. **B** Analysis of the creeping distance of housefly larvae*.* Abbreviations: Lb, Luria–Bertani medium; Cu, CuSo_4_; K, *K. pneumoniae* solution; CuK, Mixed solution of *K. pneumoniae* and CuSO_4_; P, *K. pneumoniae* phage; CuP, Mixed solution of *K. pneumoniae* phage and CuSO_4_. Asterisks indicate significant differences at *p < 0.05, **p < 0.01

In order to observe the effect of metal Cu^2+^ on *M.domestica* larvae from the whole aspect of *K. pneumoniae*, we examined their crawling ability. The results showed that feeding the mixture of CuSO_4_—*K. pneumoniae* phage could significantly inhibit the creeping ability of *M.domestica* larvae, and feeding CuSO_4_—*K. pneumoniae* could significantly promote the creeping ability of *M.domestica* larvae (Fig. 2A and B).

### Analysis of intestinal damage in *M. domestica* larvae

**Fig. 3:**
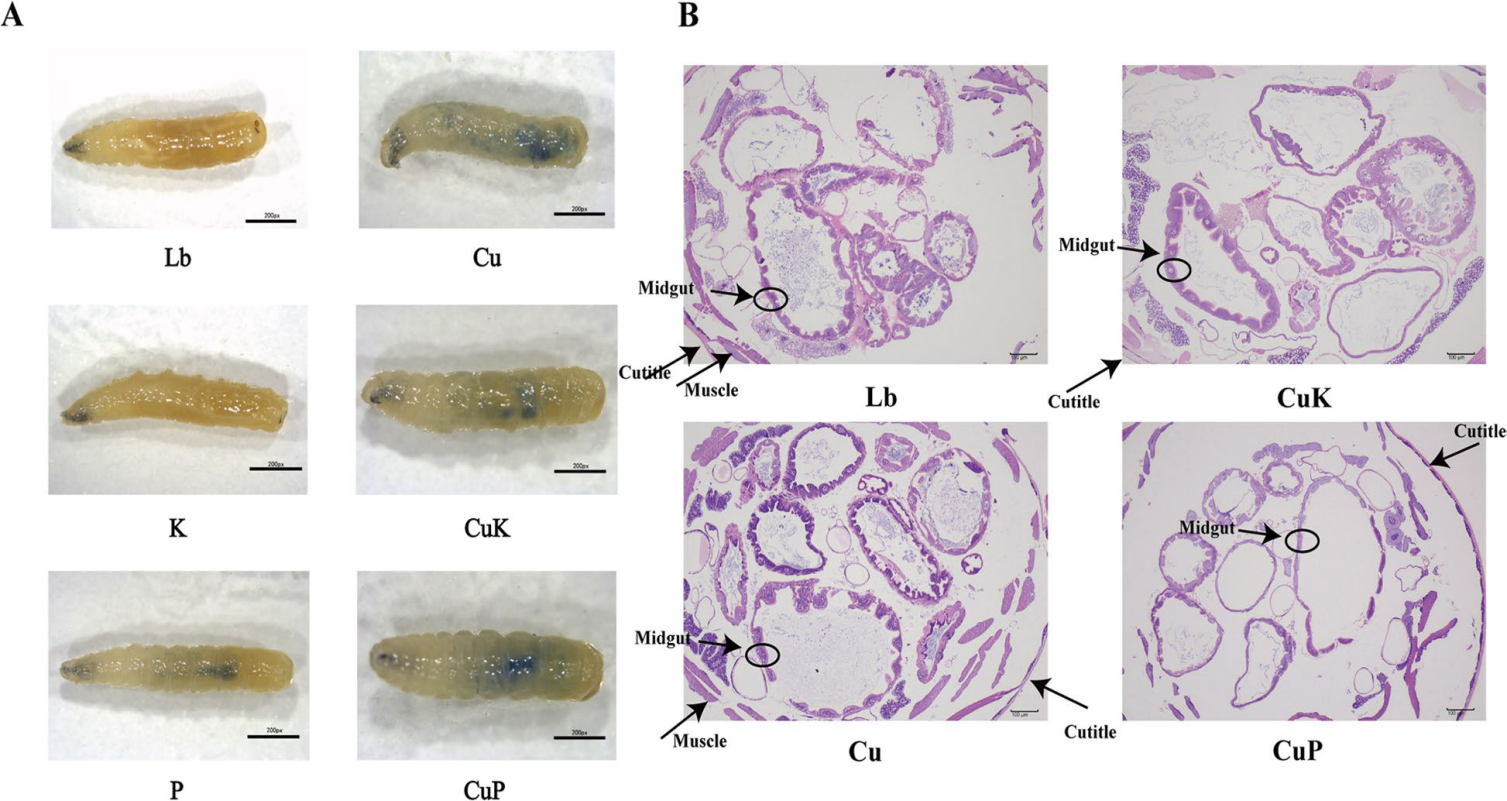
Intestinal damage of housefly larvae fed by different groups. **A** Positive trypan blue staining was present in the Cu, CuK, P, and CuP groups. No signs of tissue damage were observed in the Lb and K larvae. **B** Damaged tissue sections of domestic fly larvae in different experimental groups (100 ×). Abbreviations: Lb, Luria–Bertani medium; Cu, CuSo_4_; K, *K. pneumoniae* solution; CuK, Mixed solution of *K. pneumoniae* and CuSO_4_; P, *K. pneumoniae* phage; CuP, Mixed solution of *K. pneumoniae* phage and CuSO_4_. Asterisks indicate significant differences at *p < 0.05, **p < 0.01, ***p < 0.001,****p < 0.0001

In order to verify more visually that metallic Cu^2+^ causes damage to intestinal tissues and whether the addition of *K. pneumoniae* attenuates this damage, we conducted trypan blue staining experiments. Meanwhile, the damaged domestic fly larvae under different application conditions were observed through tissue sections. Trypan blue staining was used to identify intestinal cell damage in *M.domestica* larvae exposed to Cu^2+^- *K. pneumoniae* and its phage mixture. Positive trypan blue staining was only observed in Cu group, CuK group, P group, CuP group including indicating that these treatments induced gut damage in *M.domestica* larvae (Fig. 3A).In the intestinal tissue sections of *M. domestica* larvae, we could obviously observe that the intestinal mucosa of *M. domestica* larvae in Cu group was irregular, while this phenomenon was significantly alleviated in CuK group (Fig. 3B).

### Analysis of the microbial diversity index of *Musca domestica* larvae under the interaction of Cu^2+^—*K. pneumoniae* and its phage mixture

**Fig. 4:**
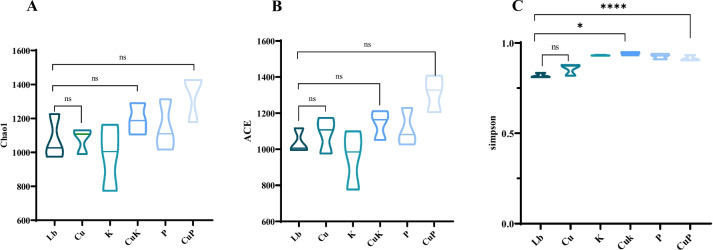
Violin plot of Microbial Species Richness (**A**, **B**) and Species Diversity (**C**) indices of *Musca domestica* larvae exposed to different experimental groups (**A**) ACE index, (**B**) Chao1 index, (**C**) Shannon index. Abbreviations: Lb, Luria–Bertani medium; Cu, CuSO_4_; K, *K. pneumoniae* solution; CuK, mixed solution of *K. pneumoniae* and CuSO_4_; P, *K. pneumoniae* phage; CuP, mixed solution of *K. pneumoniae* phage and CuSO_4._ Asterisks indicate significant differences at *p < 0.05, **p < 0.01, ***p < 0.001,****p < 0.0001

In order to study the mechanism of the anti-metal ability of *K. pneumoniae* on the growth and development of *M.domestica* larvae, we used 16S rRNA gene sequencing technology to analyze the changes in gut flora, and then verified it at the molecular level. Firstly, a total of 966,413 high-quality reads were measured from the original data after mass filtration. On the basis of 99% sequence homology, 61,480 OTUs were detected in all samples. The α-diversity of the intestinal microbiota was estimated by the community diversity index (Shannon) and richness index (ACE, Chao1) for the six groups. The results of the analysis using ACE and Chao1 index showed that there was no significant difference in microbial richness among the experimental groups compared with Lb, however, in the CuP group, the Chao1 index increased, and the intestinal microflora richness of *M.domestica* larvae increased (Fig. 4A). B). The Shannon as indicators of diversity in the OTUs in samples, indicated that there was no significant difference in the intestinal microflora diversity of *M.domestica* larvae between the Cu and CuK groups and the Lb groups; however, in the P and CuP groups, the Shannon index increased the intestinal flora diversity of *M.domestica* larvae (Fig. 4C).

### Analysis of the composition and structure of the gut flora of *Musca domestica* larvae under the interaction of Cu^2+^—*K. pneumoniae* and its phage mixture

**Fig. 5:**
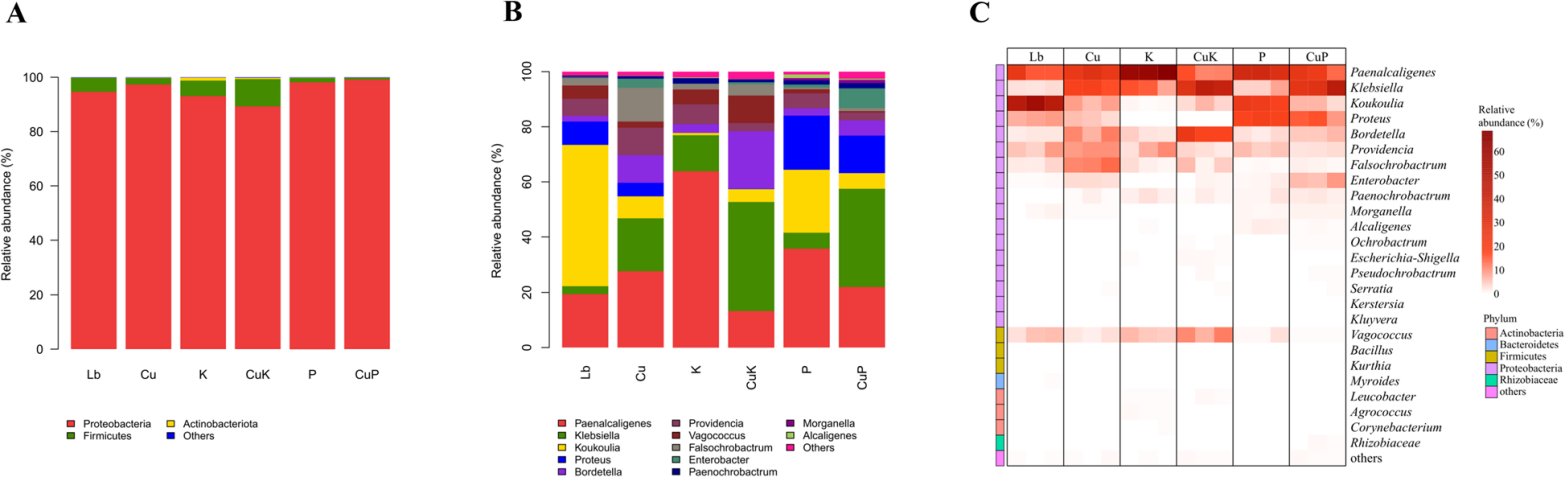
Relative abundances of bacterial components in samples of housefly larvae fed different feeds. **A** The relative abundances of bacteria at the *phylum* level*.* **B** The relative abundances of bacteria at the *genus* level. **C** Heatmaps of the relative abundances and distributions of bacterial genera in housefly larvae. Heatmaps are based on the composition of bacterial genera of the different feeding groups, with each genus color coded, as shown in the panel. Abbreviations: Lb, Luria–Bertani medium; Cu, CuSo_4_; K, *K. pneumoniae* solution; CuK, Mixed solution of *K. pneumoniae* and CuSO_4_; P, *K. pneumoniae* phage; CuP, Mixed solution of *K. pneumoniae* phage and CuSO_4_

To analyze the influence of the Cu^2+^—*K. pneumoniae* and its phage mixture on the gut microbiota of *M.domestica* larvae. The gut community structure of the larvae in different groups was analyzed. At the phylum level, *Proteobacteria* was the dominant intestinal flora in all samples. The abundance of *Proteobacteria* in the Cu group (99.44%) was higher than that in the Lb group, and that in the CuK group (98.29%) was lower than that in the Lb group and K group. When fed *Klebsiella* phage, the abundance of *Proteobacteria* increased (98.15%), and that of the CuP group (99.12%) increased and was higher than that of the Lb group, Cu group and P group (Fig. 5A). At the genus level, the community structure of gut microflora in the different groups was significantly different from that in the Lb groups. We found that compared with the Lb group, *Klebsiella* and *Enterobacter* in the intestinal tract of *M.domestica* larvae in the Cu group increased, while the relative abundance of *Koukoilia* and *Proteus* decreased significantly. In the CuK group, we found that compared with the Lb group and Cu group, the abundance of *Klebsiella* and *Bordetella* in the intestine of *M.domestica* larvae increased significantly, while compared with the K group, the abundance of *Klebsiella* and *Enterobacter* increased, and the relative abundance of *Paenalcaligenes* and *Provincia* decreased. In the CuP group, compared with those in the Lb and Cu groups, the abundances of *Enterobacter* and *Klebsiella* increased significantly. Compared with those in the P group, the abundances of *Enterobacter*, *Klebsiella* and *Bordetella* increased significantly, and the relative abundances of *Paenalcaligenes*, *Koukoilia*, *Proteus* and *Provincia* decreased (Fig. 5B). This is consistent with the heatmap analysis, through the analysis, we found that in the Cu group, *Klebsiella* and *Enterobacter* increased significantly, but in the CuK group, the *Klebsiella* increased more significantly, in the Group P, the abundance of *Klebsiella* bacteria decreased significantly, while in the CuP group, *Klebsiella* and *Enterobacter* increased significantly (Fig. 5C).

**Fig. 6:**
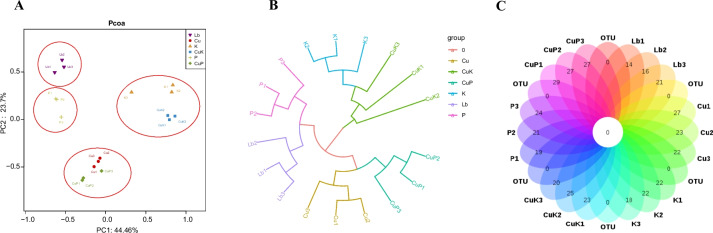
Differences in bacterial community structures and relationships between the feeding groups. **A** Principal coordinate analysis (PCoA) of bacterial community structures of the six groups. Each symbol represents one sample of intestinal bacteria. **B** Unweighted pair group method with arithmetic mean (UPGMA) evolutionary tree analysis of samples. **C** Venn diagram analysis of unique and shared OTUs of the intestinal bacteria in housefly larval samples. The number represents the number of unique OTUs in each sample and common OTUs shared by two or more samples. Abbreviations: Lb, Luria–Bertani medium; Cu, CuSO_4_; K, *K. pneumoniae* solution; CuK, mixed solution of *K. pneumoniae* and CuSO_4_; P, *K. pneumoniae* phage; CuP, mixed solution of *K. pneumoniae* phage and CuSO_4_

We further analyzed the structural differences of the gut microflora in the different samples. Principal co-ordinate analysis (PCoA) showed that the gut microflora structure of *M.domestica* larvae every group was significantly different compare with Lb groups. The gut flora in the K and CuK groups clustered together, the Cu group and CuP group clustering together, and the gut flora in the Lb group and P group clustered together separately (Fig. 6A). UPGMA tree analysis provides further evidence to support the cluster analysis of different samples (Fig. 6B). The Venn diagram revealed the common and unique OTUs in all samples, the Venn diagrams also showed the differences in gut microflora samples of *M.domestica* larvae (Fig. 6C).

### Analysis of the intestinal flora interaction of *M. domestica* larvae under the interaction of Cu^2+^—*Klebsiella pneumoniae* and its phage mixture

**Fig. 7:**
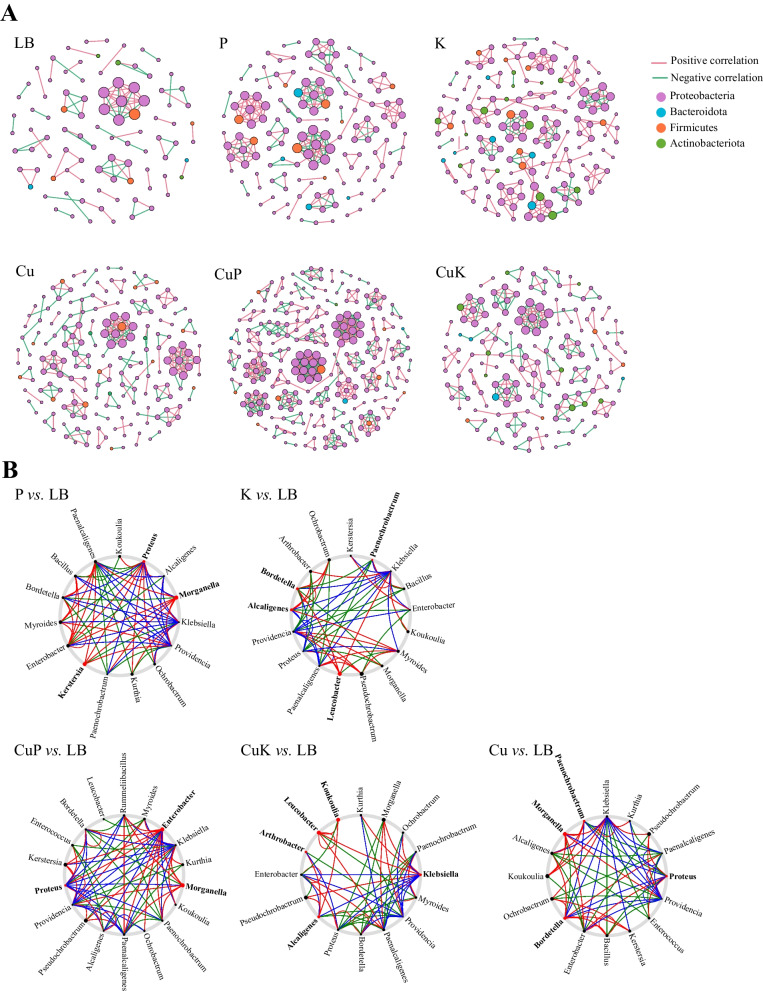
Intestinal bacterial cooccurrence microbiome networks between different processing groups. **A **Network analysis of different groups. Each point in the figure represents a species, and species with correlations are connected by a line. Red lines represent positive correlations, green lines represent negative correlations, and the intensity of the line represents the level of correlation. **B** NetShift analysis of different groups. Potential “driver taxa” of infection based on bacterial network analysis of the experimental group (P, K, CuP, CuK, and Cu) and the control groups (Lb), marked as P vs. Lb, K vs. Lb, CuP vs. Lb, CuK vs. Lb, respectively. Node sizes are proportional to their scaled NESH (neighbor shift) score (a score identifying important microbial taxa of microbial association networks), and those nodes colored red were important driver taxa. As a result, large red nodes denote particularly important driver taxa under different conditions. Line colors indicate node (taxa) connections as follows: red edges, association present only in experimental groups; green edges, association present only in control groups; blue edges, association present in both the experimental and control groups. Abbreviations: Lb, Luria–Bertani medium; Cu, CuSO_4_; K, *K. pneumoniae* solution; CuK, mixed solution of *K. pneumoniae* and CuSO_4_; P, *K. pneumoniae* phage; CuP, mixed solution of *K. pneumoniae* phage and CuSO_4_

To study the effect of feeding in different groups on the interactions of gut microflora of *M.domestica* larvae, we first constructed a related network of *M.domestica* larvae gut microflora (Fig. 7). The results showed that the CuK group significantly changed the interaction between the intestinal flora of silkworm larvae. Compared with the Lb group, the total number of nodes of the interaction network in the intestinal microflora of the Cu group increased, the average path distance increased, the average aggregation decreased, the positive correlation intensity decreased, the negative correlation intensity increased, and the negative correlation increased, which decreased the interaction stability of the intestinal microflora of *M.domestica* larvae. In the CuK group, compared with the Lb group, the total number of nodes increased, the average path distance increased, the average clustering coefficient decreased, the positive correlation decreased, the negative correlation increased, and the stability of intestinal microflora of *M.domestica* larvae decreased; compared with the Cu group, the total number of nodes decreased, the average path distance increased, the average clustering coefficient decreased, the positive correlation increased, the negative correlation decreased, which made the interactions of gut microflora of *M.domestica* larvae more stable. In the CuP group, compared with the Lb group, the total number of nodes increased, the average path distance increased, the average clustering coefficient increased, and the positive correlation increased, but the negative correlation decreased, which made the interactions of gut microflora of *M.domestica* larvae more stable. Compared with the Cu group, the summary points increased, the average path distance increased, the average clustering coefficient increased, the positive correlation increased, and the negative correlation decreased, which made the interactions of gut microflora of *M.domestica* larvae more stable (Table 1).

**Table 1:** Co-occurrence network indices of different groups

Treatment group	Network indices						
	Total nodes	Total links	Average degree	Average path distance	Average clustering coefficient	Positive correlation	Negative correlation
Lb	93	95	2.043	1.069	0.9	59.95%	41.05%
Cu	186	267	2.871	1.396	0.84	56.18%	43.82%
K	161	250	3.106	1.913	0.83	81.6%	18.4%
CuK	170	247	2.906	1.668	0.776	58.7%	41.3%
P	142	229	3.225	1.621	0.851	65.5%	34.5%
CuP	256	572	4.469	1.99	0.915	60.84%	39.16%

*Abbreviations: Lb* Luria–Bertani medium, *Cu* CuSO_4_, *K K. pneumoniae* solution, *CuK* Mixed solution of *K. pneumoniae* and CuSO_4,_ *P K. pneumoniae* phage*, CuP* Mixed solution of *K. pneumoniae* phage and CuSO_4_

In all samples, there was a high degree of connectivity within *Proteobacteria*, especially the interactions between *Proteobacteria* (89.25%). In the Cu group, the interaction between *Proteobacteria* (91.4%) was enhanced, the interaction between *Firmicutes* (6.99%) and *Proteobacteria* was enhanced, and the interaction between *Actinobacillus* (1.61%), *Proteobacteria* and *Firmicutes* was significantly weakened. In the CuK group, compared with the Lb group and Cu group, the interaction between *Proteobacteria* (90%) was enhanced, the interaction between *Firmicutes* (2.35%) and *Proteobacteria* was weakened, and the interaction between *Actinobacillus* (5.29%), *Bacteroides* (2.35%) and *Proteobacteria* was significantly enhanced. In the CuP group, compared with the Lb group and the Cu group, the interaction between *Proteobacteria* (94.92%) was enhanced, and the interaction between *Firmicutes* (3.52%), *Bacteroides* (1.17%), *Actinobacillus* (0.39%) and *Proteobacteria* was weakened (Fig. 7A). The Netshift analysis revealed that *Proteus*, *Bordetella*, *Morganella* and *Paenochrobactrum* were the potential key bacterial groups in the Cu group. In the K group, *Koukoulia*, *Leucobacter*, *Alcaligenes* and *Klebsiella* were the potential key bacterial groups in the initial microbiomes of *M.domestica* larvae. *Koukoulia*, *Leucobacter*, *Arthrobacter*, *Klebsiella* and *Alcaligenes* were the potential key bacterial groups in the CuK group initial microbiomes of *M.domestica* larvae. In the P group, *Morganella*, *Proteus* and *Kerstersia* were the potential key bacterial groups in the initial microbiomes of *M.domestica* larvae. *Koukoulia*, *Leucobacter*, *Arthrobacter*, *Klebsiella* and *Alcaligenes* were the potential key bacterial groups in the CuP group initial microbiomes of *M.domestica* larvae (Fig. 7B).

### Analysis of electron microscope results

**Fig. 8:**
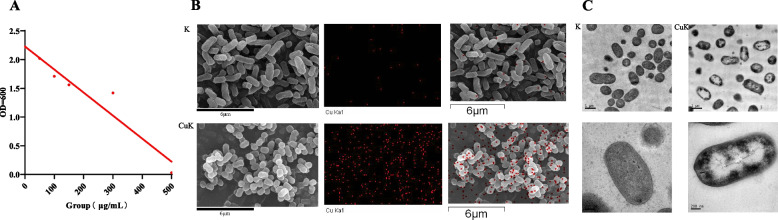
**A** *K.pneumoniae* was inoculated in different concentrations of Lb-Cu medium, and the growth of bacteria was measured under OD_600_ absorbance conditions. **B** *Scanning electron microscope* (SEM) image (10,000 ×) (**C**) *Transmission electron microscope* (TEM) image (25,000 ×). Abbreviations: K, *K. pneumoniae* solution; CuK, mixed solution of *K. pneumoniae* and CuSO_4_; CuP, mixed solution of *K. pneumoniae* phage and CuSO_4_

The in vivo model of *M.domestica* larvae showed that the addition of *K. pneumoniae* could alleviate the damage caused by Cu^2+^ to *M.domestica* larvae, We inferred that the metal adsorption of *K. pneumoniae* could reduce the effect of heavy metals on intestinal flora and then played a useful regulatory role. To verify our hypothesis, we carried out in vitro verification experiments to verify the relationship between *K. pneumoniae* and Cu^2+^. *K. pneumoniae* was cultured in different concentrations of Lb-Cu^2+^ medium (Lb-Cu medium). The results showed that in Lb-Cu medium containing different concentrations of Cu^2+^, the growth of *K. pneumoniae* was significantly inhibited with increasing Cu^2+^ concentration (Fig. 8A)*.* The scanning electron microscopy (SEM) results showed that Cu^2+^ was obviously adsorbed on *K. pneumoniae* (Fig. 8B). The cytoplasm of *K. pneumoniae* inoculated in Lb-Cu medium was obviously damaged (Fig. 8C).

### Analysis of the immune function of *M. domestica* larvae

**Fig. 9:**
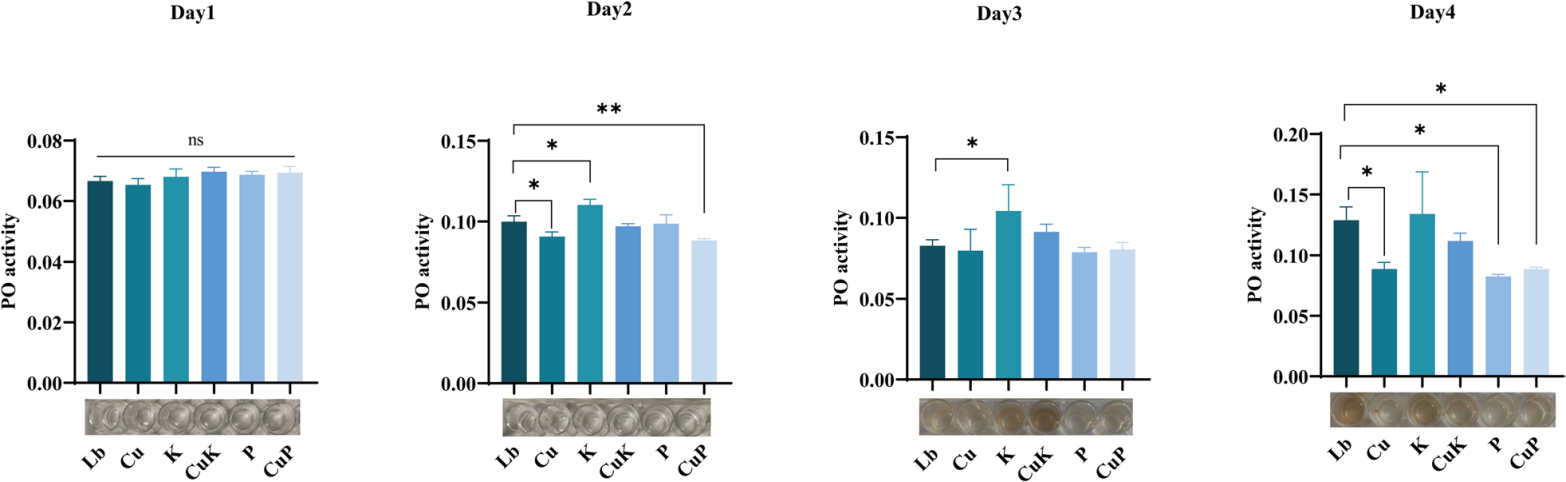
Effects of different diets on PO activity in the hemolymph of housefly larvae. Abbreviations: Lb, Luria–Bertani medium; Cu, CuSO_4_; K, *K. pneumoniae* solution; CuK, mixed solution of *K. pneumoniae* and CuSO_4_; P, *K. pneumoniae* phage; CuP, mixed solution of *K. pneumoniae* phage and CuSO_4._ Asterisks indicate significant differences at *p < 0.05, **p < 0.01

To further verify the antagonistic effect of *K. pneumoniae* on *M.domestica* larvae caused by Cu^2+^, we measured phenoloxidase (PO) activity in different experimental groups. We found that compared with the Lb group, there was no difference in phenoloxidase activity and no melanization in the hemolymph of larvae on Day 1. The hemolymph PO activity of larvae on the Day 2 for the Cu and CuP groups was significantly inhibited, and there was no melanization in larval hemolymph. There was no significant difference in PO activity between the CuK group and Lb group and no melanization in larval hemolymph. The activity of PO in the hemolymph of the larvae in the Day 4, Cu, P and CuP groups was significantly inhibited, and there was no melanization in the hemolymph of the larvae. There was no significant difference in the PO activity between the CuK group and the Lb group (Fig. 9). These results showed that the immunity of *M.domestica* larvae decreased after feeding CuSO_4_—*K. pneumoniae* alleviated the damage caused by Cu^2+^ to *M.domestica* larvae*.*

## Discussion

With the continuous development of industry, copper is used in a variety of industries. Cu is an essential metal for human, animals and plants, it is associated with numerous physiological and biochemical processe, and it acts as a cofactors in numerous enzymes such as laccase, cytochrome coxidase, polyphenol oxidase [24]. Cu is a basic micronutrient for insect respiration, pigmentation and oxidation, but it is toxic at certain exposure concentrations. In our study, when the concentration of Cu^2+^ reached 600 μg/mL, the body weight and length of *M.domestica* larvae were significantly inhibited, and death occurred. According to previous studies, when the concentration of Cu^2+^ reaches 600 μg/L, the development rate and pupation ratio of *Aedes aegypti* were significantly decreased; in addition to this, it has been shown that exposure to the nanoparticle Fe_3_O_4_ inhibits the growth and development of *M.domestica* larvae, resulting in the inability to feather properly and causing significant damage to their tissues and digestive system [25, 26]. In this study, we analyzed the alterations in the growth and development and intestinal microflora of *M.domestica* larvae under the influence of Cu^2+^—*K. pneumoniae* and its phage mixture to observe the role of the addition and removal of *K. pneumoniae* in Cu^2+^ antagonistic. We found that when Cu^2+^—*K. pneumoniae*, the damage caused by Cu^2+^ to the host was significantly reduced, while after the targeted removal of *K. pneumoniae*, the damage caused by Cu^2+^ to the host was more severe. The SEM and TEM results further explain that *K. pneumoniae* could adsorb Cu^2+^ to reduce the negative effect of Cu^2+^ on intestinal flora.

Existing studies have shown that heavy metal exposure can change the intestinal flora of insects, some researchers believe that insects can be used as biological indicators of heavy metal pollution [27]. For example, it has studies shown that heavy metals play an important impact on the intestinal flora of animals and insects such as *black soldier flies*, fish, *Amphibia,* and others [16, 28, 29]. In our study, *M.domestica* larvae were exposed to CuSO_4_, and the intestinal flora of *M.domestica* larvae changed significantly, mainly characterized by a significant increase in the abundance of *Klebsiella* and *Enterobacter*. *Enterobacter* is beneficial to *M.domestica* larval growth, which can inhibit the growth of some pathogenic strains in *M.domestica* larvae, increase the load of beneficial bacteria in the intestinal microbial community and balance the interaction of intestinal microflora in *M.domestica* larvae [30]. We assume that the increased *Enterobacter* and *Klebsiella* are the anti-damage bacteria produced by *M.domestica* larvae stimulated by Cu^2+^. *Lactobacillus* is the dominant genus of adult *Drosophila*, however, when *Drosophila* is exposed to Pb^2+^, *Komagataeibacter* becomes the main dominant genus of bacteria [31].

Beneficial bacteria play an important role in the growth and development of insects, they can participate in host metabolism, synthesize amino acids, vitamins, and nutrients, regulate hormones, physiological response, which in turn plays a regulatory role in the development process of insects [32–34]. The intestinal community diversity and humoral immunity of *M. domestica* larvae increased with the addition of *E. hormaechei, K. pneumoniae*, *Acinetobacter bereziniae* and *Enterobacter cloacae* during the growth and development of *M. domestica* larvae [35]. *Candida albicans,* a symbiotic bacterium in the intestinal tract of adult *Bactrocera minax*, it provides essential amino acids for its growth and development and can also convert urea into available nitrogen sources through metabolism, thus significantly increasing its fecundity [17]. The application of bacteria results in a significant increase in honeybee brooding, pollen and harvested honey [36]. Our study showed that after the combined application of Cu^2+^—*K. pneumoniae*, the intestinal flora of *M.domestica* larvae change significantly, the body weight and length increased significantly, and the exercise ability returned to the normal level, which indicated that the addition of *K. pneumoniae* would further increase the abundance of beneficial bacteria in the intestinal tract of *M.domestica* larvae and antagonize Cu^2+^. The results of SEM showed that *K. pneumoniae* could aggregate and adsorb Cu^2+^, alleviating the damage of Cu to other beneficial bacteria in intestinal tract, which further verified the antagonism of *K. pneumoniae* to Cu^2+^ in vivo.

Bacteriophages can target and kill pathogens without affecting other bacteria [37, 38]. In our study, when *M.domestica* larvae were fed *K. pneumoniae* phage, the abundance of *K. pneumoniae* in the intestine of *M.domestica*l arvae decreased significantly, and the intestinal community structure of *M.domestica* larvae changed. Interestingly, however, we found that the relative abundance of *K. pneumoniae* did not decrease, compared to the Lb and Cu groups after application of Cu^2+^- *K. pneumoniae* phages. The body weight and body length of *M.domestica* larvae were significantly higher than those of the Cu group. We speculate that *Klebsiella* phage may be resistant to bacteriophages and that Cu^2+^ stimulates the host to produce *Klebsiella* to fight against the damage of Cu^2+^. Zhang X et al. also found a similar phenomenon; they believe that single-use phages can establish an intestinal phage amplification model, and bacteria may soon become resistant to this phage [39], When bacteriophages are continuously added, they will not produce significant effects [40].

Beneficial bacteria not only reflect the growth and development of *M.domestica* larvae but also play an important role in antagonizing intestinal tissue damage and immunity. Related studies have shown that exposure to copper-containing compounds can lead to DNA damage, apoptosis, inflammation, changes in gene expression, tissue damage and gill damage in fish [41, 42]. In our study, we found that the intestinal tissue of *M.domestica* larvae was obviously damaged after exposure to Cu^2+^. PO is one of the necessary enzymes in the insect immune system to resist microbial invasion. PO plays an important role in the growth, development and immune function of insects [43]. Some studies have shown that when insects are invaded by exotic microorganisms, inactive prophenoloxidase (PPO) activates PO under the action of related serine proteases to form quinones. PPO can regulate phagocytosis, coating and blackening and participate in the immune defense process of insects [44]. At present, PO activity is used as a standard index to evaluate the immune ability of insects. In our study, we found that the PO activity decreased after feeding Cu^2+^ to *M.domestica* larvae, which indicated that Cu^2+^ could damage the immunity of *M.domestica* larvae. When we give Cu^2+^—*K. pneumoniae*, the PO activity of *M.domestica* larvae returned to the normal level, which indicated that the addition of *K. pneumoniae* could prevent the damage of Cu^2+^ to the immune system. After the targeted removal of *K. pneumoniae*, PO activity was more inhibited, which further confirmed the importance of *K. pneumoniae* in antagonizing the immune damage caused by Cu^2+^.

## Conclusions

In conclusion, this study revealed that *K. pneumoniae* helped *M.domestica*larvae resist damage from Cu^2+^ stress by regulating intestinal microflora and adsorbed Cu^2+^, thereby reduced Cu^2+^ effect on other bacteria (especially beneficial bacteria), thus producing obvious antagonism.

**Materials and methods**

### Materials

The houseflies used in this study were raised in the Laboratory of Vector and Vector-borne Diseases of Shandong First Medical University since 2005. Adult houseflies were fed with brown sugar and water, and the larvae were fed with wet wheat bran and milk powder [wheat bran (g): water (mL): milk powder (g) = 1:1:0.4]. Houseflies were reared in an artificial climate incubator maintained at 25 ± 1℃ and 70% relative humidity under a photo period of 12/12 h [light/dark].

*Klebsiella pneumoniae* was isolated from *M.domestica* larvae, and based on specificity, bacteriophages that could attack the bacteria were isolated [20].

A copper-containing compound (CuSO_4_) were purchased from Kaitong Chemical Reagent Institute, Tianjin, China. Copper (300 μg/mL) solution was prepared as mother liquor for follow-up experiments.

### Experiment design

*K. Pneumoniae* were inoculated into Luria–Bertani liquid (Lb liquid) and cultured at 37 °C and 110 rpm in a constant temperature culture oscillator. After 24 h of cultivation, 30 mL of *K. Pneumoniae* (OD_600_ = 2.00) stock solution was used for subsequent experiments. 30 mL of *K. pneumoniae* phage solution was diluted 10^7^-fold with Lb liquid for subsequent experiments. A total of 0.03537 g CuSO_4_⋅5H_2_O was dissolved separately in 30 mL of Lb liquid and 30 mL of *K. pneumoniae* (OD_600_ = 2.00) stock solution to prepare a CuSO_4_ solution and *K. pneumoniae* mixture, respectively. A total of 0.03537 g CuSO_4_⋅5H_2_O was dissolved separately in 30 mL of *K. pneumoniae* phage solutions to prepare CuSO_4_ and *K. pneumoniae* phage (10^7^) solution mixtures, respectively. Sterilized wheat bran (30 g) was separately mixed with 30 mL of Lb liquid medium (group Lb), CuSO_4_ solution (group Cu), *K. Pneumoniae* (OD_600_ = 2.00) (group K), CuSO_4_-*K. Pneumoniae* mixture (group CuK), *K. Pneumoniae* phage (10^7^) solution (group KP)and CuSO_4_-*K. Pneumoniae* phage mixture (group CuP) for larval feed and placed in a 5 mL sterile centrifuge tube with small holes on the top. As reported in our previous study [45], wheat bran (1.5 g-1.6 g) were added in a 1:1 proportion to the 5 mL centrifuge tube. The tubes were placed in an artificial climate incubator maintained and kept at a predetermined time point (1, 2, 3, 4 days). The same number of larvae were taken from each test tube, and their body length, body weight, pupal weight, pupation rate and emergence rate were recorded.

### Extraction of intestinal DNA and Bioinformatics analysis

DNA extraction of the intestinal bacteria and determining changes in gut composition using Illumina MiSeq Sequencing were carried out using the same method [45]. MENAP is used to analyze the OTU data of each sample, and the network was performed using Gephi (Gephi 0.10.1, France) [46–49].

### Sample preparation for scanning and transmission electron microscopy

The Luria–Bertani medium containing 300 μg/ mL Cu^2+^ of heavy metals was configured (named Lb-Cu). *K.pneumoniae* bacterial stock solution were inoculated into Lb-Cu medium and cultured for 24 h, the growth of bacteria was detected under the condition of OD_600_ absorption. Bacteria obtained by centrifugation were fixed overnight in 2.5% (v/v) glutaraldehyde. Then they were sectioned in 2 mm × 1 mm samples with parallel surfaces to get a flat surface of observation. Samples were observed using a scanning electron microscope (SEM) (Hitachi SU 8020, Tokyo, Japan). According to previous studies [49], samples were prepared and observed were observed by transmission electron microscope (TEM) (Hitachi, H-7650).

### Determination of the crawling ability of *M.domestica* larvae in different experimental groups

Three 3-day-old *Musca domestica* larvae with good growth condition were placed on solid Agar medium. The larvae were removed after 10 min, and the creeping tracks of *M.domestica* larvae were photographed and recorded and then marked with Digimizer4. The length of creeping tracks was calculated. Each experimental group was repeated 3 times.

### Trypan blue staining of *M.domestica* larvae

In this experiment, two-day-old *M.domestica* larvae were selected for staining to observe the intestinal damage of different experimental treatments to *M.domestica* larvae. Three replicates were analyzed in each group (8 larvae per replicate). The larvae of *M.domestica* in different groups crawled for 10 min to remove food traces in the gut of larvae. *M.domestica* larvae were transferred to a 1.5 mL centrifuge tube, washed thoroughly with PBS (1 ×) solution, and 1 mL of trypan blue staining solution was added and the solution was kept under shaking conditions for 30 min. The stained larvae were rinsed three times with PBS (1 ×) solution. Larval staining was eventually observed under a microscope.

### Analysis of phenoloxidase activity of *M.domestica* larvae in different experimental groups

The same number of larvae were extracted from Lb, Cu, K, CuK, P and CuP, and the phenoloxidase (PO) activity was analyzed by the same method as reported in our previous study [35].

### Statistical analysis

All data analysis was performed by IBM SPSS Statistics 20 statistical software. All data are expressed as mean ± SD. The effects of the body weight and body length of *M.domestica* larvae were compared by using Two-way ANOVA followed by Sidak correction. The microbial diversity index, pupation rate, pupal weight, eclosion, crawling distance and phenoloxidase activity in the hemolymph of the larvae were analyzed by one-way ANOVA. Significance analysis was performed by Sidak’s multiple comparisons test (*p* < 0.05).

## Data Availability

The 16 s sequence data of the microbiome were stored in the Sequence Read Archive database (BioProject accession number: PRJNA970925).
